# Influence of real-world cue exposure and mood states on drinking: testing neurobiological models of alcohol use disorder

**DOI:** 10.1007/s00213-025-06752-8

**Published:** 2025-02-10

**Authors:** Lindsay R. Meredith, Wave-Ananda Baskerville, Carrie Lee, Erica N. Grodin, Kate M. Wassum, Lara A. Ray

**Affiliations:** 1 Department of Psychology, University of California, Los Angeles, CA USA; 2 Department of Psychiatry and Biobehavioral Sciences, University of California, Los Angeles, CA USA; 3 Brain Research Institute, University of California, Los Angeles, CA USA

**Keywords:** Allostatic, Incentive-sensitization, Naturalistic reporting, Cue exposure, Negative emotionality, Alcohol use disorder, Daily diary

## Abstract

**Rationale:**

Two prominent neurobiological models of addiction, the allostatic and incentive-sensitization models, have guided clinical research on alcohol use disorder (AUD). While these models are often viewed in isolation, it is plausible these theories are complimentary.

**Objectives:**

Use naturalistic, daily diary reports to determine whether positive and negative mood states influence alcohol cue sensitivity in a clinical sample with AUD.

**Methods:**

This is an exploratory analysis of daily diary data collected from a non-treatment seeking sample with current AUD over two weeks. Eligible adult participants (*N* = 50) were enrolled in a medication trial for AUD. Each morning, participants retrospectively reported on pre-drinking mood states, alcohol cue exposure, and craving levels, and subsequent alcohol intake occurring the previous day. Multilevel models tested the singular and interactive relationships between cue exposure and mood states with craving and drinking. Within-person and between-person outcomes were assessed. Exploratory analyses examined whether individuals with withdrawal-related dysphoria were more vulnerable to mood states and cue-reactivity.

**Results:**

Greater cue exposure was associated with higher daily drinking levels (*p* = .001), but not daily alcohol craving. Higher negative mood (*p* < .0001) and lower positive mood (*p* = .012) were associated with higher daily alcohol craving, but not same-day drinking. As negative mood levels increased (*p* < .01) and positive mood levels decreased (*p* = .010), the relationship between cue exposure and same-day drinking became stronger. These findings were most pronounced among those with withdrawal-related dysphoria.

**Conclusions:**

Findings provided concomitant support for the allostatic model and incentive-sensitization model as determinants of alcohol craving and drinking among individuals with AUD.

**Supplementary Information:**

The online version contains supplementary material available at 10.1007/s00213-025-06752-8.

## Introduction

The allostatic and incentive-sensitization models are two prominent neurobiological models of addiction that have been proposed and validated through extensive preclinical research (Berridge et al. 2009; Koob et al. 1999; Robinson and Becker 1986). These models have guided clinical research and conceptualizations of alcohol use disorder (AUD), often in isolation of each other. The allostatic model explains addiction as a transition from using alcohol for its pleasurable effects, considered as positive reinforcement, to using alcohol’s relieving effects to avoid feeling bad, referred to as negative reinforcement (Koob and Le Moal 2001). With continued heavy alcohol use, homeostatic correction responses dampen reward system function and recruit brain stress systems to cause a negative emotional state to emerge in withdrawal (Koob 2015; Koob and Schulkin 2019). Addiction develops when one transitions to using alcohol to avoid this negative emotional state, which could likewise be created by other social, psychological, and environmental factors (Gallo and Matthews 2003; Mor and Winquist 2002; Taylor et al. 2004). Contrastingly, the incentive-sensitization model emphasizes the sensitization of brain rewards systems to trigger excessive alcohol “wanting” (Robinson and Berridge 2001). Consequently, alcohol-associated cues trigger intense cravings and compulsive behaviors to seek out and consume alcohol (Robinson and Berridge 2001). Both models provide theoretical explanations for the transition to addiction, such that alcohol drives progressive brain changes and impacts psychological states, cognition, and behavioral responses. Alcohol craving is a hallmark of both models (e.g., “wanting”, preoccupation/ anticipation stage), and a ubiquitous marker of addiction (King et al. 2021). Yet, these models are often viewed in isolation, and implicate distinct brain changes and, thus, treatment approaches (Koob and Le Moal 2001; Robinson and Berridge 2001).

Recent efforts have focused on translating these preclinical models into research applicable to clinical populations. For instance, cross-sectional human laboratory research testing the allostatic model showed that craving significantly correlated with alcohol-induced stimulation (e.g., feeling talkative, energized, elated) among individuals with heavy drinking patterns but not among those with AUD (Bujarski and Ray 2014), indicating a potential transition away from positive reinforcement drinking (Bujarski et al. 2018). However, while those with AUD had higher baseline negative affect, there was no significant association between negative affect and self-administration to support negatively reinforced drinking (Bujarski et al. 2018; Bujarski and Ray 2014). In capturing individual changes over time, longitudinal work following young adults showed that coping motivations predicted frequent alcohol intake among those who transitioned to AUD, while enhancement motivations predicted frequent alcohol intake regardless of AUD status (Cho et al. 2019). Similarly, acute responses of stimulation and wanting alcohol in response to alcohol administration were actually shown to increase over time, rather than decrease, among individuals with AUD (King et al. 2021). Taken together, findings provide partial support for both the allostatic and incentive-sensitization theories. Individuals who have transitioned to AUD experience a continued desire to drink for enhancement of positive affect and “wanting” for alcohol, but also increasing levels of negative affect and a desire to drink for relief from this state.

The above findings point to a need to test and compare the allostatic and incentive-sensitization models in a single study. One approach to test these theories in combination is examining the relationship between mood states and alcohol cue-reactivity. This concept has been tested through laboratory-based methods, such as the well-established alcohol cue exposure and stress exposure paradigms (Plebani et al. 2012; Sinha 2009; Snelleman et al. 2014). Evidence from the implementation of these methods supports the notion that stress exposure increases sensitivity to alcohol cues, particularly for those who report drinking to cope with negative affect. While the cue exposure paradigm has some predictive utility for alcohol intake (Lukas et al. 2013; Monti et al. 2000), it is suggested that only 50–65% of individuals with AUD display cue reactivity in this context (Plebani et al. 2012). An alternative approach is intensive micro-longitudinal monitoring of real-world data on in vivo cue exposure, craving, and positive and negative mood states (Dora et al. 2023; Votaw et al. 2022). This provides robust ecological validity, as cues in the natural environment are likely stronger triggers and reinforcers than those presented in the laboratory (Carpenter et al. 2020; Cofresí et al. 2022; Fatseas et al. 2015). Carpenter et al. (2020) argued that micro-longitudinal, naturalistic daily diary reporting offers a valuable and complementary method to clinical studies, which can increase power and ecological validity and reduce recall error. For instance, procedures assessing cue-reactivity in both the laboratory and natural environment, showed that cue-induced craving was even more pronounced when present in participants’ own environment (Miranda et al. 2014). Yet, few studies have examined the influence of cue exposure on mood or craving using these naturalistic methods.

Much more research has looked at the broader relationship among mood, craving, and alcohol use using daily diary or ecological momentary assessment (EMA) methods. While EMA provides excellent temporal precision, daily diary methods have yielded comparable results in terms of drinking estimates (Stevens et al. 2020) and estimates of emotion variability (Schneider et al. 2020). Craving captured in the natural environment may mediate the association between subjective stress and greater alcohol intake, even in the context of social drinking (Wemm et al. 2022). A meta-analysis including participant-level data from 69 studies that administered daily or momentary reports and primarily enrolled non-clinical young adult samples found that higher positive mood was associated with greater daily alcohol intake and heavy drinking; however, variations in negative mood were not consistently associated with alcohol behaviors (Dora et al. 2023). In an EMA study enrolling a clinical sample of female participants with AUD, findings demonstrated that high momentary negative affect and low momentary positive affect was associated with subsequent elevations in craving and heavy drinking (Leenaerts et al. 2024). As such, capturing natural, dynamic variations in daily affective states and craving in relation to person-specific alcohol cues across time may make studying processes related to allostasis and incentive-sensitization models more clinically relevant. Research further suggests that individual factors, such as withdrawal-related dysphoria (i.e., drinking to avoid unpleasant feelings, such as nervousness and irritability) (Grodin et al. 2024; Kady et al. 2024), coping motivations (Cho et al. 2019), and greater severity of AUD (Leenaerts et al. 2024), may strengthen these relationships and influence drinking behaviors.

To further the translation of neurobiological models of addiction to clinical samples, the present study used naturalistic, daily diary reports to probe relationships among mood states and cue-reactivity. Participants with AUD were assessed at an initial screening for the presence of withdrawal-related dysphoria, defined as drinking to avoid unpleasant feelings. Over the course of two weeks, participants reported daily on their mood states, exposure to alcohol cues, craving, and alcohol intake. To bridge the two models, we examined how within-person and between-person mood states and cue exposure influenced alcohol craving and intake in three steps. We first assessed the incentive-sensitization model by looking at daily cue-induced craving and drinking. Second, we assessed the allostatic model by examining positively and negatively reinforced craving and drinking. Third, we tested these models in combination, by determining whether daily positive and negative mood states moderated alcohol cue-sensitivity. In an exploratory fashion, we further tested the hypothesis that individuals with withdrawal-related dysphoria, a marker of allostasis, would be more vulnerable to the potential additive influence of daily alcohol cues and negative mood states on measures of craving and alcohol use. This is in line with the notion that withdrawal-related dysphoria captures negative reinforcement from alcohol use and is marked by negative emotionality.

## Methods

### Study overview

Daily diary assessment (DDA) data utilized in the present study were collected for a two-week randomized clinical trial of ibudilast for heavy drinking reduction and negative mood improvement in a sample of individuals with AUD (ClinicalTrials.gov identifier: NCT03489850). Full study procedures and primary trial findings are available (see (Grodin et al. 2021)). In addition, a published secondary analysis using this DDA data examined medication-related effects on subjective response to alcohol (Meredith et al. 2022). The present exploratory analysis sought to test the relationship among daily cue exposure and mood on craving and alcohol intake while covarying for medication effects. Following randomization, participants were asked to fill out electronic DDAs to report on previous day mood states, craving, exposure to alcohol-related cues, and subsequent alcohol use. All trial procedures were approved by the University of California, Los Angeles Institutional Review Board. Before completing study procedures, participants received a full study explanation and provided written informed consent.

### Participants

For the trial, a community-based sample of adults with current AUD was recruited. Eligibility criteria was detailed in the original trial publication (see (Grodin et al. 2021). Inclusion criteria included being between the ages of 21–50 years, meeting criteria for current DSM-5 diagnosis for AUD (First et al. 2015), reporting heavy baseline drinking patterns (> 14 drinks/week for men and > 7 drinks/week for women), and having reliable internet access for DDAs. In sum, 190 individuals attended the initial in-person screening visit, 81 were clinically eligible to complete a physical screening, and 52 participants were randomized to study medication (*n* = 24) or placebo (*n* = 28). In the present study, DDA data from 50 participants were analyzed, as these participants completed at least one assessment following randomization.

### Screening procedures

Study procedures took place at an outpatient research clinic housed in an academic medical center. The screening process consisted of an initial telephone-screening interview, an in-person behavioral screening visit, and a physical screening visit to assess medical eligibility for the trial. Prior to starting visits, participants needed to show a breath alcohol concentration of 0.00 g/dl and urine toxicology test negative for all illicit substances, excluding cannabis. Participants who met all eligibility criteria were invited to attend a randomization visit where they were randomly assigned to receive either 50 mg twice daily of ibudilast or matched placebo, stratified by sex and presence of withdrawal-related dysphoria.

### Study assessments

#### Baseline assessments

At the baseline screening visit, participants completed interviews and surveys to capture individual differences and screen for eligibility criteria. These assessments included items on demographic characteristics (e.g., age, sex, race, income), alcohol and substance use patterns and history, and psychological functioning and DSM-5 diagnoses. Sample characteristics are detailed in previous report focused on DDAs from this trial (Meredith et al. 2022). The Reasons for Heavy Drinking Questionnaire (Adams et al. 2016) (RHDQ) was administered to measure participants’ motivations for heavy drinking. As established by the parent trial (Grodin et al. 2021), the presence of withdrawal-related dysphoria was determined as follows: raw scores on the RHDQ question #6: “I drink because when I stop, I feel bad (I am nervous, irritable, and I sleep poorly)”, which ranged from 0–10, were dichotomized based on a cut-off of > 6 points (yes vs. no). Thus, we sought to capture whether participants “agreed” with this statement that they drink to relieve negative emotionality.

#### Daily diary assessments

Participants received daily morning reports at 8 AM for two weeks. Each morning report remained available to participants until the following day’s report was sent out. These electronic DDAs were sent via text message or email and asked participants to retrospectively report on previous day experiences. For each DDA, participants were first asked whether they drank alcohol the prior day. If they reported that they drank alcohol the prior day, they were prompted to indicate yesterday’s drink types and quantity consumed. Subsequently, they completed ratings of mood states, alcohol craving, and exposure to alcohol-related cues *before* they started drinking ‘yesterday’, which allowed us to assess the potential effect of cues on subsequent same-day alcohol intake. If participants did not consume alcohol the prior day, they were similarly prompted to complete ratings of mood states, alcohol craving, and exposure to alcohol-related cues generally throughout the prior day (i.e., ‘yesterday’). Data from both drinking and non-drinking days were included in the analyses. To capture cue exposure, participant were asked (Yes or No), “Did you see any of the following yesterday?” or “Before you drank yesterday, did you see any of the following?” and were presented with a list of eight cues: (1) alcohol ads/ picture, (2) drinking on TV, (3) a liquor store, (4) a bar, (5) a drinking place other than a bar, (6) other people drinking, (7) people I usually drinking with, and (8) places where I usually drink.

The Profile of Mood States—Short Form (POMS-SF) survey was used to measure daily positive and negative mood states (Curran et al. 1995; McNair et al. 1971). POMS-SF is a validated rating scale which captures several dimensions of transient mood states via a 5-point Likert scale. To keep participant burden low while completing DDAs, only certain items from POMS-SF were included and asked, “How did you feel yesterday (before drinking)?” Specifically, the following items were used for the POMS scales: Negative Mood (downhearted, discouraged, uneasy, and anxious) and Positive Mood (joyful, cheerful, energetic, and lively). The correlation between positive and negative mood scales on non-drinking days was r = −0.38 and before any drinking the association was r = −0.46). Interclass correlation estimates derived from unconditional models for the positive and negative mood scales were 0.52 and 0.51, respectively, representing moderate reliability. Alcohol craving was assessed by a single-item urge rating asking, “How strong was your urge to drink alcohol yesterday?” (No Urge = 0 to Strongest Ever = 10; Ray et al. 2007, 2010). Quantity of standard drinks was based on established guidelines and entries were verified by staff.

### Data analytic plan

Descriptive and statistical analyses were completed in SAS Version 9.4. To create a daily negative mood state score, select items from POMS-SF depression and tension subscales were summed (range 0—16), in line with previous reports (Bujarski et al. 2015; Meredith et al. 2022; Sheets et al. 2015). Similarly, to create a daily positive mood state score, select items from the vigor subscale were summed (range from 0—16). A daily total cue type exposure variable was created to capture one’s degree of exposure to types of alcohol cues, in which the number of cue types seen was summed (range 0 – 8).

The first set of models tested whether greater daily exposure to alcohol-relevant cue types was associated with same-day increases in craving level and number of drinks consumed (primary model equations included in Supplemental File 1). In these models, we also tested whether alcohol-relevant cues would be associated with changes in average craving and average number of drinks consumed during the two-week period. Our next set of models tested whether greater daily positive or negative mood state was associated with same-day changes in craving level and number of drinks consumed. In these models, we also tested whether mood would be associated with changes in average craving and average number of drinks consumed during the two-week period. Next, we tested the interactive effect of positive or negative mood state and cue-exposure on same-day drinking by including a two-way interaction of mood × cue exposure. Finally, we tested the interactive effect of withdrawal-related dysphoria, positive or negative mood, and cue exposure on same-day drinking by including a three-way cross-level interaction of these variables (withdrawal-related dysphoria × mood × cue exposure). In all models, level-2 covariates included were biological sex (male or female; binary), age (continuous) and medication condition (ibudilast or placebo; binary). Day of study (i.e., 1 – 14) was included as a covariate in all models to account for potential changes in drinking behavior over the two-week period. Notably, medication condition was not a significant predictor in any models tested. Both drinking and non-drinking days were included in all models, as restricting the analyses to only drinking days could bias the results (e.g., number of drinking days varied among participants). This also allowed us to capture the relationship between affect and craving when no drinking takes place, which was common among participants in this sample (~ 40% of reports consisted of non-drinking days).

As this data comes from a micro-longitudinal trial with repeated measures, multilevel models with random intercepts were conducted to account for clustering of observations within participants. In line with best practices, level 2 covariates were centered at the grand mean (CGM; (Enders and Tofighi 2007). The focal statistical predictors, cue exposure, negative mood, and positive mood, were centered at the grand mean as well as within cluster (CWC; group mean) to examine both between and within-person effects (Bauer et al. 2006); within-person, day-level effects were of primary interest for models testing moderators of drinking. Missing data were accounted for by using restricted maximum likelihood estimation (REML), which is suitable to account for data missing at random (McNeish 2017; Heisig and Schaeffer 2019). Log transforming drinking outcomes to improve normality did not meaningfully alter findings.

## Results

### Participant characteristics

Participants were 50 non-treatment seeking individuals with current AUD who completed at least one DDA during the two-week trial (% meeting for mild, moderate, and severe AUD as follow: 24%, 46%, 30%). The average age was 32.7 years, 66% reported their sex as male, and 68% reported having annual household incomes < $60,000. In addition, the majority of the sample identified their race as White (56%), 14% as Black or African American, and 12% as mixed race (see (Meredith et al. 2022) for full details). Approximately one quarter of the sample identified their ethnicity as Hispanic or Latino/a/x. On average, participants consumed alcohol on 22 days (75%) and had 5.6 drinks per drinking day in the month prior to screening; 54% reported smoking cigarettes and 30% had a positive urine screen for THC.

### Daily diary assessment descriptives

Completion rate for DDAs was 92.6% (data available for 653 DDAs). Participants were missing between 0 to 4 days of reports over two weeks and completed an average of 13.06 (*SD* = 1.14) DDAs, with 7.92 being drinking days and 5.14 being non-drinking days. On average, participants completed reports within 4 h of receiving the notification (median completion time = 2 h). Estimated means for cue exposure, mood, craving, and drinks per day are presented in Table 1. These estimates account for missing data and clustering observations within person.

**Table 1:** Estimated means for cue exposure, mood, craving, and daily alcohol use

	Total sample (*N* = 50)	Participants without withdrawal dysphoria (*N* = 31)	Participants with withdrawal dysphoria (*N* = 19)
Variable	Mean (SE)	Mean (SE)	Mean (SE)
Positive mood^a^	6.08 (0.38)	6.45 (0.49)	5.49 (0.59)
Negative mood^a^	2.67 (0.32)	2.29 (0.35)	3.31 (0.60)
Urge^b^	3.01 (0.28)	2.33 (0.26)	4.12 (0.52)
Cue Exposure^c^	3.13 (0.24)	3.20 (0.29)	3.03 (0.44)
Daily number of drinks^d^	3.52 (0.41)	2.75 (0.30)	4.78 (0.90)

Estimated means drawn from unconditional models to account for missing and nested data; ^a^ subscale drawn from the Profile of Mood States- Short Form, possible range: 0 - 16; ^b^single-item Urge rating, possible range 0 - 10; ^c^cue exposure based on count of total cue types seen in given day, possible range 0 - 8; ^d^ average number of drinks per day according to daily diary reports (range 0 – 26).

### Relationship between cue exposure and craving

The slope estimate indicated that daily cue exposure was associated with higher same-day craving levels, but this was not statistically significant after accounting for covariates, *b* = 0.07 (*SE* 0.05), *p* = 0.108. Similarly, greater average cue exposure was associated with higher average craving during the two-week period, but this was not statistically significant, *b* = 0.29 (*SE* 0.17), *p* = 0.094. Neither medication condition, age, nor study day were significant statistical predictors in *any* of the following models.

### Relationship between cue exposure and drinking

**Fig. 1:**
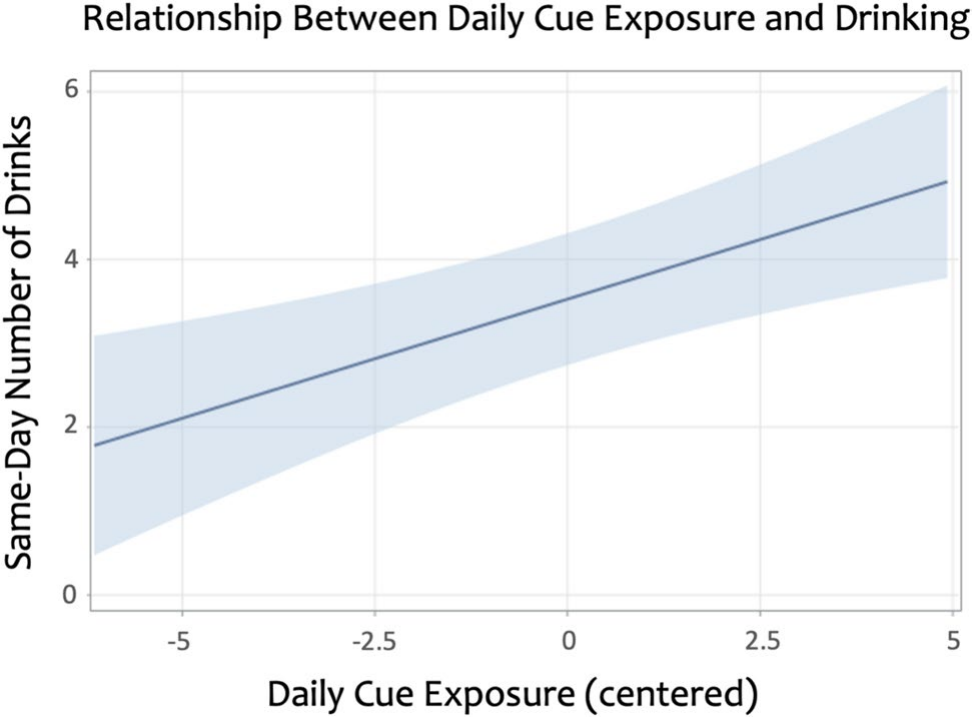
Naturalistic reporting among individuals with alcohol use disorder showed that within-person daily alcohol cue exposure was significantly associated higher same-day alcohol intake

After accounting for covariates, daily cue exposure was significantly associated with same-day number of drinks, *b* = 0.28 (*SE* 0.09), *p* = 0.001, such that greater number of cue types seen was associated with higher daily drinking levels (see Fig. 1). This tells us that we would expect a 0.28-point increase in same-day drinks for each additional cue type seen, relative to one’s own average. Average level of cue exposure was not significantly associated with drinking during the two-week period, *b* = 0.31 (*SE* 0.24), *p* = 0.208.

### Relationship between mood and craving

#### Negative mood

**Fig. 2:**
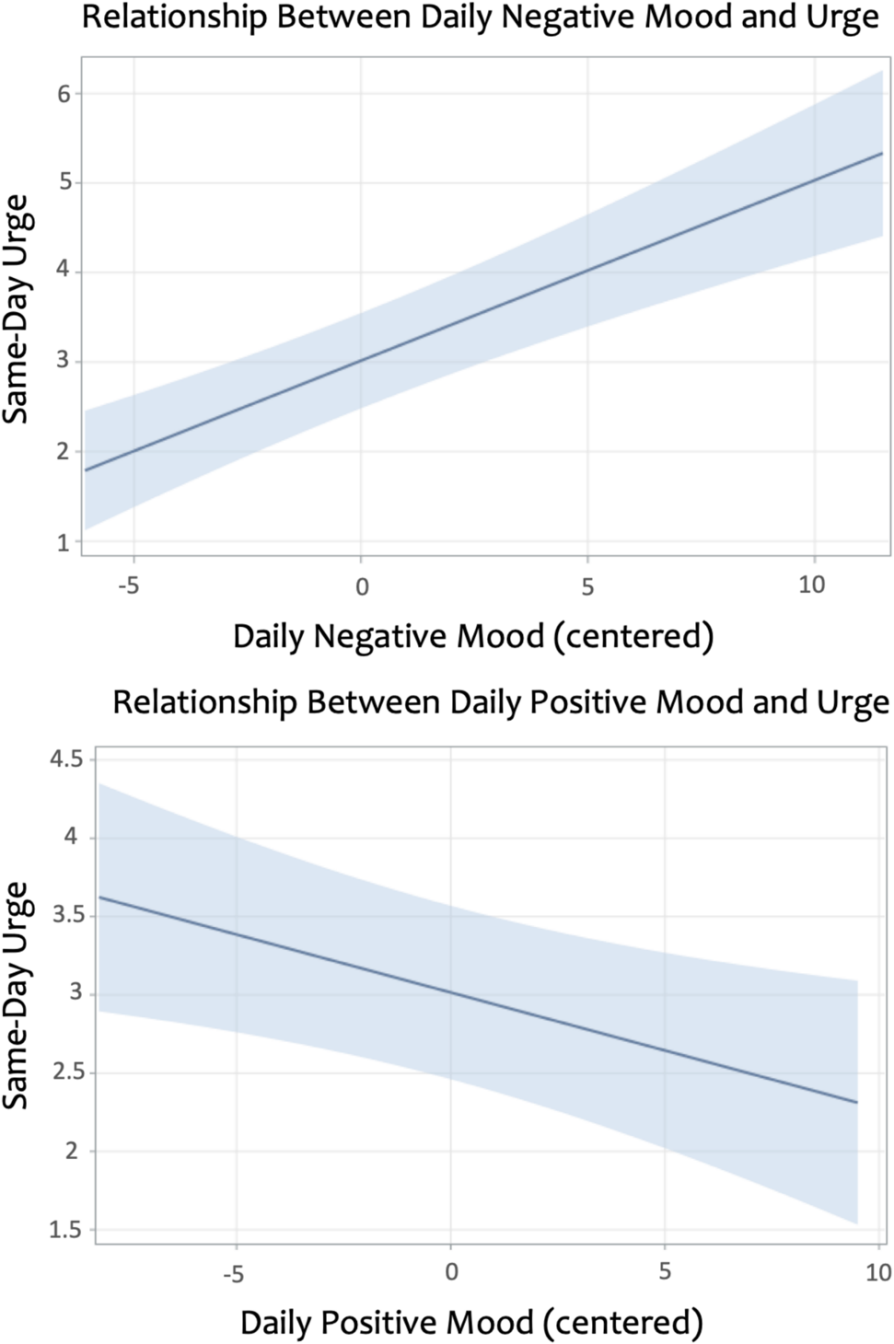
Naturalistic reporting among individuals with alcohol use disorder showed that within-person daily negative mood (top panel) and daily positive mood (bottom panel) were significantly associated with same-day alcohol craving

After accounting for covariates, daily negative mood was significantly associated with same-day craving levels, *b* = 0.20 (*SE* 0.03), *p* < 0.0001, in which higher negative mood was associated with higher daily craving (see Fig. 2). This tells us that we would expect a 0.20-point increase in same-day craving for each one-point increase in negative mood, relative to one’s own average. Similarly, higher average level of negative mood was significantly associated with higher craving during the two-week period, *b* = 0.30 (*SE* 0.12), *p* = 0.019. This tells us that we would expect a 0.30-point increase in average drinking for each one-point increase in negative mood.

#### Positive mood

After accounting for covariates, daily positive mood was significantly associated with same-day craving levels, *b* = −0.07 (*SE* 0.03), *p* = 0.012, such higher positive mood was associated with lower daily craving or lower positive mood was associated with higher daily craving (see Fig. 2). This tells us that we would expect a 0.07-point increase in same-day craving for each one-point decrease in positive mood, relative to one’s own average. Average level of positive mood was not significantly associated with craving levels during the two-week period, *b* = −0.15 (*SE* 0.11), *p* = 0.163.

### Relationship between mood and drinking

#### Negative mood

Higher negative mood was associated with higher daily drinking, although after accounting for covariates, this did not reach the significance threshold, *b* = 0.12 (*SE* 0.07), *p* = 0.064. Average level of negative mood was significantly associated with higher drinking levels during the two-week period, *b* = 0.36 (*SE* 0.18), *p* < 0.05. This tells us that we would expect a 0.36-point increase in average drinking for each one-point increase in negative mood.

#### Positive mood

After accounting for covariates, daily positive mood was not significantly associated with same-day number of drinks, *b* = 0.09 (*SE* 0.06), *p* = 0.092, although same-day drinking tended to increase with more elevated positive mood. Average level of positive mood was similarly not significantly associated with drinking during the two-week period, *b* = −0.15 (*SE* 0.15), *p* = 0.317.

### Interactive relationship between daily mood, cue exposure, and drinking

#### Negative mood

**Fig. 3:**
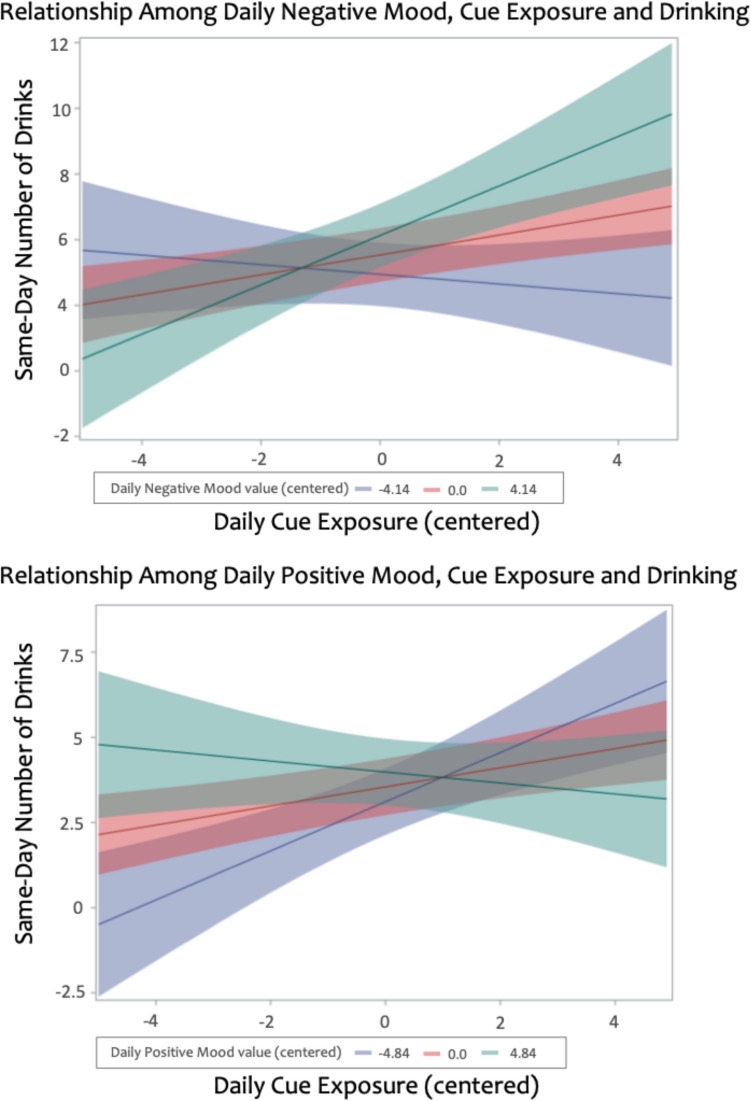
Naturalistic reporting among individuals with alcohol use disorder showed that within-person daily negative mood (top panel) and positive mood (bottom panel) significantly moderated the association between cue exposure and same-day alcohol intake. Visualization shows interaction probed for mood values at the mean and 2 SDs above and below mean

A significant two-way interaction among daily negative mood, cue exposure, and same-day drinking was detected, *b* = 0.11 (*SE* 0.04), *p* < 0.01. This interaction was probed at values of negative mood: mean, ± 1 SD from mean, and ± 2 SDs from mean, which allowed a comparison of cue exposure simple slope estimates at varying levels of negative mood (see Table 2). Findings showed that on days where an individual had higher than typical daily negative mood (+ 1 or 2 SDs above mean), the relationship between cue exposure and same-day drinking was most pronounced (*p’s* < 0.001), compared to lower than typical negative mood (*p’s* > 0.40; see Fig. 3). As such, with increasing daily negative mood levels, the relationship between cue exposure and same-day drinking became stronger.

**Table 2:** Simple slope estimates for models on the interactive relationship between daily mood, cue exposure, and drinking

Models predicting same-day drinking			Full sample (*N* = 50)		
	Slope estimate for cue exposure	Standard error	Degrees of freedom^a^	*t-*value	*p*-value
Negative mood value					
2 SDs below mean	−0.15	0.19	608.7	−0.78	0.439
1 SD below mean	−0.08	0.12	604.2	0.65	0.519
Mean	0.30	0.09	597.0	3.50	0.0005**
1 SD above mean	0.53	0.12	604.8	4.23	< 0.0001***
2 SDs above mean	0.75	0.20	608.8	3.82	0.0001**
Positive mood value					
2 SDs below mean	0.72	0.19	606.5	3.72	< 0.001**
1 SD below mean	0.50	0.12	603.1	4.06	< 0.0001***
Mean	0.28	0.09	596.9	3.23	0.001**
1 SD above mean	0.06	0.12	602.8	0.50	0.620
2 SDs above mean	−0.16	0.19	606.4	−0.85	0.397

^a^ Kenward-Rogers degrees of freedom were utilized to reduce bias and obtain more accurate *p*-value estimates; * indicates a significant positive relationship between daily cue exposure and number of drinks at given values of daily mood (centered within-person; ** = *p* < 0.001, *** = *p* < 0.0001)

#### Positive mood

A significant two-way interaction among daily positive mood, cue exposure, and same-day drinking was detected, *b* = −0.09 (*SE* 0.04), *p* = 0.010. This interaction was probed at values of positive mood: mean, ± 1 SD from mean, and ± 2 SDs from mean (see Table 2). Findings showed the opposite effect as above, such that on days where an individual had lower than typical positive mood (- 1 or 2 SDs below mean), the relationship between cue exposure and same-day drinking was most pronounced (*p*’s < 0.001), compared to higher than typical daily positive mood (*p’s* > 0.35; see Fig. 3). For example, with decreasing daily positive mood levels, the relationship between cue exposure and same-day drinking became stronger.

### Exploratory analyses: interactive relationship between daily mood, cue exposure, withdrawal-related dysphoria and drinking

#### Negative mood

A significant three-way interaction between negative mood, cue exposure, and withdrawal-related dysphoria was detected, *b* = 0.23 (*SE* 0.07), *p* < 0.001. When probing based on presence of withdrawal-related dysphoria, findings show a significant two-way interaction of negative mood × cue exposure on drinking among participants endorsing withdrawal-related dysphoria (n = 19;* b* = 0.25 (*SE* 0.09), *p* = 0.005), but not among those without this marker (n = 31; *b* = 0.05 (*SE* 0.05), *p* = 0.239). Consistent with above, the relationship between cue exposure and same-day drinking became stronger with increasing daily negative mood levels among those with withdrawal dysphoria, compared to lower than typical daily negative mood (see Table 3). In the full model only, biological sex was significantly associated with alcohol use, *b* = −1.74 (*SE* 0.82), *p* = 0.038, where males reported higher levels of drinking over two weeks.

**Table 3:** Simple slope estimates for participants with withdrawal-related dysphoria: exploratory models on the interactive relationship between daily mood, cue exposure, and drinking

Models predicting same-day drinking			Participants with withdrawal-related dysphoria (*n* = 19)		
	Slope estimate for cue exposure	Standard error	Degrees of freedom^a^	*t*-value	*p*-value
Negative mood value					
2 SDs below mean	−0.71	0.38	225.3	−1.88	0.062
1 SD below mean	−0.17	0.24	224	−0.73	0.469
Mean	0.37	0.17	222	2.12	0.035*
1 SD above mean	0.91	0.26	224.3	3.52	< 0.001***
2 SDs above mean	1.45	0.41	225.4	3.56	< 0.001***
Positive mood value					
2 SDs below mean	1.09	0.37	225	2.94	0.004**
1 SD below mean	0.71	0.24	223.9	2.93	0.004**
Mean	0.33	0.18	222	1.87	0.063
1 SD above mean	−0.05	0.23	223.7	−0.23	0.820
2 SDs above mean	−0.43	0.36	224.9	−1.21	0.227

^a^ Kenward-Rogers degrees of freedom were utilized to reduce bias and obtain more accurate *p*-value estimates; * indicates a significant positive relationship between daily cue exposure and number of drinks at given values of daily mood (centered within-person) for participants with withdrawal-related dysphoria (* = *p* < 0.05, ** = *p* < 0.01, *** = *p* < 0.001)

#### Positive mood

A significant three-way interaction between positive mood, cue exposure, and withdrawal-related dysphoria was also detected, *b* = −0.16 (*SE* 0.05), *p* = 0.003. When probing based on presence of withdrawal-related dysphoria, findings show a significant two-way interaction of positive mood × cue exposure among participants endorsing withdrawal-related dysphoria, *b* = −0.16 (*SE* 0.07), *p* = 0.015, but not among those without this marker, *b* = −0.03 (*SE* 0.04), *p* = 0.521. Consistent with positive mood effects above, the relationship between cue exposure and same-day drinking became stronger with decreasing daily positive mood levels among those with withdrawal dysphoria, compared to higher than typical positive mood (see Table 3). In the full model only, biological sex was significantly associated with alcohol use, *b* = −1.83 (*SE* 0.81), *p* = 0.029, where males reported higher levels of drinking over two weeks.

## Discussion

The neurobiological understanding of addiction has progressed rapidly in the past two decades, yet opportunities for translation to clinical samples remain (Heilig et al. 2016; Nieto et al. 2021). Towards translation of neurobiological models, the present study sought to test hypotheses derived from the combination of incentive-sensitization (Robinson and Berridge 2001) and allostatic models (Koob and Le Moal 2001) of addiction in a clinical sample of individuals with AUD. Specifically, we tested the relative contribution of emotionality (i.e., mood ratings) and incentive salience (i.e., cue-reactivity) on subjective craving for alcohol and drinking outcomes using naturalistic reports over a two-week period. Overall, findings from this study showed that cue exposure and mood states were significantly associated with same-day craving and alcohol intake. Furthermore, mood states moderated the relationship between cue exposure and daily drinking.

The incentive sensitization model of addiction proposed that alcohol-associated cues trigger intense urges to seek out and consume alcohol (Robinson and Berridge 2001). Accordingly, in this study greater exposure to a range of alcohol-related cue types was associated with heavier levels of same-day drinking on the individual level. However, this did not extend to average levels of cue exposure, as between-person effects of cue exposure on drinking over two weeks were not significant. This demonstrates the benefits of naturalistic reports measuring day-to-day fluctuations in one’s environment (Carpenter et al. 2020). Interestingly, while cue exposure was positively related to alcohol craving, this association was not significant. Previous research has suggested that craving and reduced inhibitory control may serve as mediators between cue exposure and elevations in alcohol intake (Field and Jones 2017; Green et al. 2019). Yet, craving was not tested as a mediator in our models given the temporal nature of the design (i.e., cue exposure and craving assessed concurrently). Our earlier laboratory work had suggested alcohol-induced craving was lower among individuals with AUD (Bujarski and Ray 2014). Thus, it is critical to establish whether cue-induced craving is consistently sustained in AUD samples compared to those with heavy drinking only, particularly in real-world settings. In summary, these results provided support for the incentive-sensitization model by demonstrating that alcohol cues were meaningfully associated with alcohol intake among individuals with current AUD.

The allostasis model proposed that addiction develops when one transitions to using alcohol to avoid the negative emotional state created by withdrawal or other social, psychological, and environmental factors (Koob 2015; Koob and Schulkin 2019; Gallo and Matthews 2003; Mor and Winquist 2002; Taylor et al. 2004). Accordingly, the present analysis supported a robust effect of negative mood on craving, such that higher negative mood was associated with greater same-day craving. Other naturalistic reporting studies enrolling adults with AUD (Leenaerts et al. 2024), including for alcohol treatment (Votaw et al. 2022), have found similar within-person associations. As noted above, a comprehensive meta-analysis on the relationship between daily affect and alcohol use concluded that across individuals with a range of drinking patterns, they were not more likely to drink on days they experienced high negative affect (Dora et al. 2023). In line with the current project, these researchers highlighted the need for daily diary studies specifically enrolling adults meeting criteria for AUD. In this context, we found that negative affect was more directly associated with daily craving than same-day drinking. Average levels of negative mood were also associated with higher levels of both craving and drinking over the two-week period. This relationship is reliably found in the literature in which progression toward severe AUD is associated with higher levels of negative mood, craving, and drinking (Ehlers et al. 2019; Pavkovic et al. 2018; Thorberg et al. 2019).

Continuing, high positive mood did not promote alcohol craving or drinking in this study and instead a lack of positive affect might have enhanced a desire to drink, consistent with a recent EMA report (Leenaerts et al. 2024). These findings are in contrast to previous research primarily focused on younger samples with heavy drinking, which support enhancement motivation or high positive affect leading to greater alcohol intake (Dora et al. 2023). Nevertheless, it is plausible that enhancement and coping motives can coexist and even interact (Mann et al. 2018). For instance, using the same dataset, we found that these participants do experience the stimulating effects of acute alcohol intake, suggesting the rewarding effects of alcohol remain (Meredith et al. 2022). Similarly, longitudinal studies evidence the persistence of alcohol-induced stimulation in samples with AUD (King et al. 2016, 2014). Perhaps negative reinforcement processes can co-exist with positive reinforcement, or alcohol-induced enhancement, in the phenomenology of AUD.

The results discussed thus far provide concomitant support for the incentive sensitization model and for the allostatic model in this sample. To extend this, our analyses also allowed for interactions between model constructs, revealing unique ways in which emotionality and incentive salience may operate synergistically. Mood states moderated the relationship between cue-exposure and drinking, such that on days when participants were exposed to multiple alcohol-related cue types and had higher negative mood, they engaged in the heaviest rates of same-day drinking. However, experiencing high negative mood without exposure to alcohol cues was associated with much lower same-day drinking. Thus, cue exposure may promote drinking in the face of negative affect, which is supported by laboratory studies combining negative mood (i.e., stress induction) and alcohol cue-exposure paradigms (Ray 2011; Sinha et al. 2009; Snelleman et al. 2014). A complimentary inverse pattern emerged for positive mood such that on days when participants were exposed to multiple alcohol-related cues and had lower positive mood, they engaged in the heaviest rates of same-day drinking. In this case, participants may be seeking to enhance positive affect through alcohol’s stimulating properties. This speaks to the complexity of understanding and treating AUD, in which internal triggers, such as negative mood states may exacerbate the risk of external triggers, such as environmental or social cues, leading to heavy drinking or relapse.

A final set of analyses interrogate the role of negatively reinforced drinking motivations, which was an a-priori randomization variable in this study defined as the presence or absence of drinking to relieve withdrawal-related dysphoria. Findings that cue exposure moderated the relationship between mood states and drinking were most salient among individuals with withdrawal dysphoria compared to those without it. Individuals with withdrawal-related dysphoria may be more at risk for mood-dependent drinking and cue-induced drinking, in line with previous research (Dyar & Kaysen, 2023; Snelleman et al. 2014). When these two mechanisms come together in additive fashion, individuals with withdrawal-related dysphoria may be at greatest risk for heavy drinking and possibly relapse. In other words, risk mechanisms associated with the allostatic model (i.e., negative emotionality) and incentive sensitization (i.e., cue-reactivity) appear to be complementary in explaining alcohol use among individuals with AUD, particularly those reporting withdrawal-related dysphoria versus more reward-driven drinking. These insights could inform personalized medicine approaches to care, if replicated in future samples. Providers could feasibly assess for one’s drinking motives (i.e., reward vs. relief drinking) during intake appointments through a one-question item. Although more burdensome, daily dairies could be implemented in a treatment context to better assess triggers for heavy drinking to inform a functional analysis or coping skills plan (Litt et al. 2024). These types of patient reports are implemented in cognitive behavioral therapy for insomnia (CBT-I) through daily sleep diaries, which guide behavioral interventions, such as sleep restriction and stimulus control.

While these findings are encouraging and fill gaps in the translation of addiction neurobiology to clinical samples, caution is warranted in considering study limitations. To that end, ratings of mood and craving were collected retrospectively and at the same time, whereas EMA methods allow for a more granular analysis of time course and dose-dependent alcohol responses (Ray et al. 2010). Therefore, EMA methods may better capture momentary associations between cue exposure and alcohol craving. The real-world context of the study comes at the expense of experimental control over assessments and lack of bioverification of alcohol use levels. While drinking levels were not associated with study day in our models, participation in a research study may promote reductions in drinking or other behavioral changes from baseline (Baskerville et al. 2021); for instance, participants drank 75% of days prior to screening but 60% of days during the 14-day diary period. Participants in this study were non-treatment seeking, and not actively trying to change their drinking, meaning these findings may not generalize to more severe or treatment-seeking samples (Ray et al. 2017). In treatment-seeking AUD populations, it may be worthwhile to explore other drinking outcomes, such as any alcohol use. While a large number of observations per participant were included and advanced statistical methods employed, the sample size for individuals with withdrawal-related dysphoria was low (*n* = 19). The relative impact and salience of various cue types, such as contextual cues, social cues, or media cues remains an open question in need of further investigation. It may be beneficial to record and track person-specific cues in future studies. This study used a count of “cue type”, which did not allow us to differentiate the relative impact of the cue-type nor the potential incremental impact of exposure to multiple same-type cues (e.g., repeatedly seeing people drinking on TV or in person). Assessing the relative salience of each alcohol cue type would enhance our understanding of their impact on subjective craving and alcohol use. Taken together, limitations suggest that additional studies be undertaken using complementary methods, such as EMA and longitudinal designs in large samples of individuals with AUD and comorbid conditions.

In closing, this naturalistic study enrolling a clinical sample of AUD advances translational science of addiction by simultaneously testing hypotheses from the incentive sensitization model and the allostatic model. Findings provided concomitant support for the role of negative emotionality (i.e., allostatic model) and exposure to alcohol cues (i.e., incentive-sensitization model) as determinants of craving and alcohol use in individuals with current AUD. Interestingly, exploratory analyses using real-world daily reporting, found evidence of synergy between the two models, in which mood states moderated the relationship between cue exposure and same-day drinking. On days when individuals were exposed to multiple alcohol-related cues and had higher negative mood or lower positive mood, they engaged in the heaviest rates of same-day drinking. Furthermore, this pattern of results was strongest among individuals reporting using alcohol to alleviate negative affect/withdrawal-related dysphoria. More research capturing daily fluctuations in mood and cue exposure using intensive micro-longitudinal assessment in larger samples with AUD, such as with a multi-burst EMA design over time (Edney et al. 2023), is needed to corroborate and extend these exploratory findings. Information garnered from future work on this topic could enable researchers to better understand individual AUD trajectories in the context of these dynamic processing (e.g., cue-reactivity, stress-induced drinking) and inform neurobiological models of addiction. These methods could further be implemented over the course of alcohol treatment and recovery (Levinson et al. 2024).

Risk mechanisms associated with the allostatic model and incentive-sensitization model appear to be complementary in explaining alcohol use in individuals with AUD, particularly those reporting withdrawal-related dysphoria. The synergistic effects of cue-induced and mood-induced mechanisms of craving and alcohol use speak to the centrality of these neurobiological models in clinical samples. These results may inform personalized medicine approaches to AUD care. This study implemented important translational neuroscience principles by constructing hypotheses from neurobiological models of addiction and applying them with human clinical samples (Nieto et al. 2021).

## Supplementary Information

Below is the link to the electronic supplementary material.

Supplementary Material 1.

## Data Availability

The data that support the findings of this research are available from authors upon reasonable request.
