# Correction to: Infuence of real-world cue exposure and mood states on drinking: testing neurobiological models of alcohol use disorder

**DOI:** 10.1007/s00213-025-06769-z

**Published:** 2025-03-04

**Authors:** Lindsay R. Meredith, Wave-Ananda Baskerville, Carrie Lee, Erica N. Grodin, Kate M. Wassum, Lara A. Ray

**Affiliations:** 1 Department of Psychology, University of California, Los Angeles, CA USA; 2 Department of Psychiatry and Biobehavioral Sciences, University of California, Los Angeles, CA USA; 3 Brain Research Institute, University of California, Los Angeles, CA USA

**Correction to: Psychopharmacology**

10.1007/s00213-025-06752-8

In the published paper, the 2nd author’s name (Wave-Ananda Baskerville) is now incorrect — with an added Mann on the end of the last name.

Correct 2nd author last name: Baskerville.

The original article has been corrected.

