# Supplementary material for: Influence of real-world cue exposure and mood states on drinking: testing neurobiological models of alcohol use disorder
Source: Psychopharmacology (Berl). 2025 Feb 10;242(8):1727–39. doi: 10.1007/s00213-025-06752-8 (PMC12296835; doi:10.1007/s00213-025-06752-8)
Supplement: Supplementary file 1 — Supplementary Material 1. [file 213_2025_6752_MOESM1_ESM.docx]

**Supplemental File 1.** **Fitted Regression Model Equations for Primary Multilevel Analyses**

$$Y_{ij}=\left( \beta_{0}+ b_{0j} \right)+ \beta_{1}X_{1ij}^{cwc}+ \beta_{2}X_{2ij}^{cgm}+ \beta_{3}X_{3j}^{cgm}+ \beta_{4}X_{4j}^{cgm}+\beta_{5}X_{5j}^{cgm}+ \beta_{6}X_{6i} + \varepsilon_{ij}$$

$${DailyNumDrinks}_{ij}=\left( \beta_{0}+ b_{0j} \right)+ \beta_{1}{Cues}_{1ij}^{cwc}+ \beta_{2}{Cues}_{2ij}^{cgm}+ \beta_{3}{Sex}_{3j}^{cgm}+ \beta_{4}{Condition}_{4j}^{cgm}+\beta_{5}{Age}_{5j}^{cgm}+ \beta_{6}{Day}_{6i}+ \varepsilon_{ij}$$

$${DailyCraving}_{ij}=\left( \beta_{0}+ b_{0j} \right)+ \beta_{1}{Cues}_{1ij}^{cwc}+ \beta_{2}{Cues}_{2ij}^{cgm}+ \beta_{3}{Sex}_{3j}^{cgm}+ \beta_{4}{Condition}_{4j}^{cgm}+\beta_{5}{Age}_{5j}^{cgm}+ \beta_{6}{Day}_{6i}+ \varepsilon_{ij}$$

$${DailyNumDrinks}_{ij}=\left( \beta_{0}+ b_{0j} \right)+ \beta_{1}{PosMood}_{1ij}^{cwc}+ \beta_{2}{PosMood}_{2ij}^{cgm}+ \beta_{3}{Sex}_{3j}^{cgm}+ \beta_{4}{Condition}_{4j}^{cgm}+\beta_{5}{Age}_{5j}^{cgm}+ \beta_{6}{Day}_{6i}+ \varepsilon_{ij}$$

$${DailyCraving}_{ij}=\left( \beta_{0}+ b_{0j} \right)+ \beta_{1}{PosMood}_{1ij}^{cwc}+ \beta_{2}{PosMood}_{2ij}^{cgm}+ \beta_{3}{Sex}_{3j}^{cgm}+ \beta_{4}{Condition}_{4j}^{cgm}+\beta_{5}{Age}_{5j}^{cgm}+ \beta_{6}{Day}_{6i}+ \varepsilon_{ij}$$

$${DailyNumDrinks}_{ij}=\left( \beta_{0}+ b_{0j} \right)+ \beta_{1}{NegMood}_{1ij}^{cwc}+ \beta_{2}{NegMood}_{2ij}^{cgm}+ \beta_{3}{Sex}_{3j}^{cgm}+ \beta_{4}{Condition}_{4j}^{cgm}+\beta_{5}{Age}_{5j}^{cgm}+ \beta_{6}{Day}_{6i}+ \varepsilon_{ij}$$

$${DailyCraving}_{ij}=\left( \beta_{0}+ b_{0j} \right)+ \beta_{1}{NegMood}_{1ij}^{cwc}+ \beta_{2}{NegMood}_{2ij}^{cgm}+ \beta_{3}{Sex}_{3j}^{cgm}+ \beta_{4}{Condition}_{4j}^{cgm}+\beta_{5}{Age}_{5j}^{cgm}+ \beta_{6}{Day}_{6i}+ \varepsilon_{ij}$$

$$Y_{ij}=\left( \beta_{0}+ b_{0j} \right)+ \beta_{1}X_{1ij}^{cwc}+ \beta_{2}X_{2ij}^{cwc}+ \beta_{3}X_{3j}^{cgm}+ \beta_{4}X_{4j}^{cgm}+\beta_{5}X_{5j}^{cgm}+ \beta_{6}X_{6i}+ \beta_{7}(X_{1ij}^{cwc})(X_{2ij}^{cwc})+\varepsilon_{ij}$$

$${DailyNumDrinks}_{ij}=\left( \beta_{0}+ b_{0j} \right)+ \beta_{1}{Cues}_{1ij}^{cwc}+ \beta_{2}{NegMood}_{2ij}^{cwc}+ \beta_{3}{Sex}_{3j}^{cgm}+ \beta_{4}{Condition}_{4j}^{cgm}+\beta_{5}{Age}_{5j}^{cgm}+ \beta_{6}{Day}_{6i}+ \beta_{7}({Cues}_{1ij}^{cwc})({NegMood}_{2ij}^{cwc})+ \varepsilon_{ij}$$

$${DailyNumDrinks}_{ij}=\left( \beta_{0}+ b_{0j} \right)+ \beta_{1}{Cues}_{1ij}^{cwc}+ \beta_{2}{PosMood}_{2ij}^{cwc}+ \beta_{3}{Sex}_{3j}^{cgm}+ \beta_{4}{Condition}_{4j}^{cgm}+\beta_{5}{Age}_{5j}^{cgm}+ \beta_{6}{Day}_{6i}+ \beta_{7}({Cues}_{1ij}^{cwc})({PosMood}_{2ij}^{cwc})+ \varepsilon_{ij}$$

**Note**. Superscript of ‘cgm’ indicates variable was centered at the grand mean, while ‘cwc’ indicates variable was centered within cluster (person-specific mean); this centering allowed us to examine both between-person and within-person effects

**Supplemental Material for Article:** Influence of Real-World Cue Exposure and Mood States on Drinking: Testing Neurobiological Models of Alcohol Use Disorder.

Journal: Psychopharmacology

Authors: L. R. Meredith, W. A. Baskerville, C. Lee, E. N. Grodin, K. M. Wassum, & L. A. Ray
